# Coronary Sinus Neuropeptide Y Levels and Adverse Outcomes in Patients With Stable Chronic Heart Failure

**DOI:** 10.1001/jamacardio.2019.4717

**Published:** 2019-12-26

**Authors:** Olujimi A. Ajijola, Neal A. Chatterjee, Matthew J. Gonzales, Jeffrey Gornbein, Kun Liu, Dan Li, David J Paterson, Kalyanam Shivkumar, Jagmeet P. Singh, Neil Herring

**Affiliations:** 1 Neurocardiology Research Center of Excellence, Cardiac Arrhythmia Center, University of California, Los Angeles; 2 Massachusetts General Hospital, Boston; 3 Department of Biomathematics, University of California, Los Angeles; 4 British Heart Foundation Centre of Research Excellence, Department of Physiology, Anatomy, and Genetics, Burdon Sanderson Cardiac Centre, University of Oxford, Oxford, England

## Abstract

**Importance:**

Chronic heart failure (CHF) is associated with increased sympathetic drive and may increase expression of the cotransmitter neuropeptide Y (NPY) within sympathetic neurons.

**Objective:**

To determine whether myocardial NPY levels are associated with outcomes in patients with stable CHF.

**Design, Setting, and Participants:**

Prospective observational cohort study conducted at a single-center, tertiary care hospital. Stable patients with heart failure undergoing elective cardiac resynchronization therapy device implantation between 2013 and 2015.

**Main Outcomes and Measures:**

Chronic heart failure hospitalization, death, orthotopic heart transplantation, and ventricular assist device placement.

**Results:**

Coronary sinus (CS) blood samples were obtained during cardiac resynchronization therapy (CRT) device implantation in 105 patients (mean [SD] age 68 [12] years; 82 men [78%]; mean [SD] left ventricular ejection fraction [LVEF] 26% [7%]). Clinical, laboratory, and outcome data were collected prospectively. Stellate ganglia (SG) were collected from patients with CHF and control organ donors for molecular analysis. Mean (SD) CS NPY levels were 85.1 (31) pg/mL. On bivariate analyses, CS NPY levels were associated with estimated glomerular filtration rate (eGFR; *r_s_* = −0.36, *P* < .001); N-terminal–pro hormone brain natriuretic peptide (*r_s_* = 0.33; *P* = .004), and LV diastolic dimension (*r_s_* = −0.35; *P* < .001), but not age, LVEF, functional status, or CRT response. Adjusting for GFR, age, and LVEF, the hazard ratio for event-free (death, cardiac transplant, or left ventricular assist device) survival for CS NPY ≥ 130 pg/mL was 9.5 (95% CI, 2.92-30.5; *P* < .001). Immunohistochemistry demonstrated significantly reduced NPY protein (mean [SD], 13.7 [7.6] in the cardiomyopathy group vs 31.4 [3.7] in the control group; *P* < .001) in SG neurons from patients with CHF while quantitative polymerase chain reaction demonstrated similar mRNA levels compared with control individuals, suggesting increased release from SG neurons in patients with CHF.

**Conclusions and Relevance:**

The CS levels of NPY may be associated with outcomes in patients with stable CHF undergoing CRT irrespective of CRT response. Increased neuronal traffic and release may be the mechanism for elevated CS NPY levels in patients with CHF. Further studies are warranted to confirm these findings.

**Trial Registration:**

ClinicalTrials.gov identifier: NCT01949246

## Key Points

**Question:**

Is the adrenergic cotransmitter neuropeptide Y (NPY) associated with outcomes in patients with stable heart failure (HF)?

**Findings:**

In a cohort of patients with stable HF undergoing cardiac resynchronization therapy device implantation, coronary sinus blood was sampled for NPY levels. A threshold level of NPY was identified, which was associated with death, heart transplant, and ventricular assist device placement; molecular studies on human sympathetic neurons indicated increased release of NPY in HF patients.

**Meaning:**

Using NPY, hyperadrenergic activation associated with adverse outcomes may be identifiable in patients with stable HF.

## Teaser

This cohort study examines whether myocardial neuropeptide Y levels in coronary sinus blood samples are associated with outcomes in patients with stable chronic heart failure.

## Introduction

The autonomic nervous system critically regulates the normal heart, although in diseased conditions, its adverse remodeling contributes to the pathophysiology.^1^ Increased cardiac adrenergic signaling is associated with cardiac dysfunction and risk of death,^2^ while reduced parasympathetic drive is observed in the failing heart.^3^ As such, biomarkers of adrenergic activity are of significant interest in mortality risk stratification.^4^

Cardiac sympathetic nerve terminals release several neurotransmitters including catecholamines (predominantly norepinephrine), galanin, and neuropeptide Y (NPY).^5^ Circulating catecholamines predict risk of death in patients with chronic heart failure (HF)^6^; however, it is unknown whether NPY is associated with adverse outcomes in chronic HF. Neuropeptide Y, which has a longer half-life, is an important modulator of cardiovascular function,^7,8^ promotes vasoconstriction,^9^ reduces parasympathetic activity,^10^ and increases myocyte calcium loading,^11^ all of which may be detrimental.

We examined coronary sinus (CS) NPY levels in a prospective cohort of patients with stable CHF at the time of elective cardiac resynchronization therapy (CRT) device implantation, during which the CS is readily accessible. Coronary sinus blood was chosen for sampling over peripheral venous blood to avoid the potential contaminating effect of NPY from other tissues beds (eg, gastrointestinal tract). We aimed to determine whether CS NPY levels are associated with (1) adverse outcomes in patients with stable left ventricular (LV) dysfunction and (2) CRT response. In a similar cohort of HF patients, stellate ganglion neurons, which predominantly provide adrenergic innervation to the heart and a major source of cardiac NPY, were also examined and compared with neurons in control patients (organ donors) to examine mechanisms underlying elevated CS NPY levels.

## Methods

### Study Population

Study approval was obtained from the Massachusetts General Hospital institutional review board. Data were obtained from patients enrolled in the prospective, single-center, observational, Biomarkers to Predict CRT Response in Patients with CHF (BIOCRT) study. Consecutive patients were enrolled between September 2013 and January 2015, and all patients gave written informed consent. Patients were not chosen and were not enrolled only if exclusion criteria were met. The inclusion and exclusion criteria for the BIOCRT study are detailed in the eMethods in the Supplement.

### Blood Sampling and NPY Assay

During device implantation, blood was drawn from a guide catheter at the CS ostium. The sample was allowed to clot for 15 minutes and centrifuged immediately at 1500 × g for 5 minutes. Samples were aliquoted and stored at −80°C until use. Deidentified serum samples were assayed for NPY levels at the University of California, Los Angeles, Immune Assessment Core Laboratory, using an enzyme-linked immunosorbent assay for NPY (EZHNPY-25K; EMD Millipore) according to manufacturer's instructions. The interassay and intra-assay coefficients of variation for the NPY assay in this study were less than 8.1% and less than 6.1%, respectively.

### Clinical Outcomes

Major adverse cardiovascular events (MACE) were defined as death, cardiac transplant (OHT), or ventricular assist device (VAD) placement. Heart failure hospitalization was considered an additional outcome. The CRT response was determined by the HF clinical composite score, with a CRT responder defined by improved score from baseline to 6-month follow-up, assessed from clinical records by 2 blinded cardiologists, and a third if discrepant categorization occurred. The stellate ganglia immunohistochemistry and quantitative polymerase chain reaction methods are detailed in the eMethods in the Supplement.

### Statistical Analysis

Mean (SD) or median/interquartile range are reported with *P* values computed via the *t* test or Mann-Whitney test, respectively. Associations between continuous predictors and NPY were assessed using the Spearman correlation (*r_s_*) and spline/linear regression.

Continuous NPY vs time to MACE was assessed via Cox regression and by finding the best threshold separating low from high MACE hazard via recursive partitioning, ie, the first split of a survival tree, which finds the split where the (log) hazard rate ratio is maximally far from 1.0 (log hazard rate maximally far from zero). This approach does not make an a priori assumption about a specific cutoff value or whether there is such a value. This partitioning was performed controlling for age, reduced glomerular filtration rate (GFR), and LV ejection fraction (LVEF). These covariates were selected by virtue of being risk factors for MACE, independent of NPY. Variables, such as hypertension, hydralazine use, and diabetes mellitus with or without insulin use, were not adjusted for because they affect NPY levels and hence are not risk factors for outcomes independent of NPY.

For stellate ganglion neuronal studies, data are presented as mean (SD). Control and cardiomyopathy patients were compared using a Welch *t* test and 2-tailed analysis of variance for normally distributed data, and the Mann-Whitney or Kruskal-Wallis test for data not normally distributed. Statistical significance was indicated at a 2-sided *P* value less than .05. Analysis was performed using GraphPad Prism (GraphPad). Additional statistical methods are detailed in the eMethods in the Supplement.

## Results

At the time of CRT implantation, 105 patients underwent CS blood sampling. Demographics and baseline characteristics are summarized in Table 1. Mean (SD) age in the cohort was 68 (12) years, 82 were men (78%), and mean (SD) LVEF was 26% (7%). Patients were optimized with β-blockers (95 of 105 [90%]); angiotensin-converting enzyme inhibitor, angiotensin receptor blocker, or nitrate/hydralazine combination (100 of 105 [95%]); and/or an aldosterone antagonist (26 of 105 [25%]) prior to CRT implantation.

**Table 1.** Baseline Characteristics of Study Participants

Patient Characteristic	No./Total No. (%)
Age, mean (SD), y	68 (12)
Male	82/105 (78)
White race/ethnicity	100/105 (95)
BMI, mean (SD)	29 (6)
ICM	54/105 (51)
Cardiovascular disease risk factors	
Hyperlipidemia	74/105 (70)
Diabetes mellitus	37/105 (35)
Hypertension	77/105 (73)
Tobacco use history	57/105 (54)
Medications	
β-Blocker	95/105 (90)
ACE inhibitor	62/105 (59)
ARB	22/105 (21)
Spironolactone	26/105 (25)
Nitrate	29/105 (28)
Hydral	5/105 (5)
Statin	74/105 (70)
Diuretic	80/105 (76)
Aspirin	79/105 (75)
Renal function, mean (SD), mg/dL	
BUN	28 (16)
Cr	1.36 (0.52)
eGFR	56.5 (19.6)
Cardiac function, mean (SD)	
LVEF, %	26 (7)
LVIDd, mm	53 (10)
LVEDV, mL	224 (80)
Clinical status	
NYHA functional class	
I	0/105
II	27/105 (26)
III	74/105 (70)
IV	4/105 (4)
MQOL score, mean (SD)	35 (24)
6MWT, mean (SD)	892 (374)
ECG	
QRS width, mean (SD), ms	164 (23)
NSR	67/105 (63.80)
Paced	21/104 (20.2)
Afib	16/104 (15.4)
BP, mean (SD), mm Hg	
Systolic	116 (14)
Diastolic	68 (9)
HR, mean (SD), bpm	71 (11)
CRT-D	99/105 (94)

Abbreviations: ACE, angiotensin-converting enzyme inhibitor; ARB, angiotensin receptor blocker; BMI, body mass index (calculated as weight in kilograms divided by height in meters squared); BP, blood pressure; bpm, beats per minute; BUN, blood urea nitrogen; Cr, creatinine; CRT-D, cardiac resynchronization therapy defibrillator (as opposed to CRT pacemaker); ECG-Afib, atrial fibrillation on electrocardiogram; ECG-NSR, normal sinus rhythm on electrocardiogram; ECG Paced, presence of ventricular pacing on electrocardiogram; eGFR, estimated glomerular filtration rate; HR, heart rate; ICM, ischemic cardiomyopathy; LVEDV, left ventricular end diastolic volume; LVEF, left ventricular ejection fraction; LVIDd, left ventricular internal diameter in diastole; MQOL, Minnesota Quality of Life Score; 6MWT, 6-minute walk test; NYHA, New York Heart Association.

SI conversion factor: To convert creatinine to micromoles per liter, multiply by 88.4.

### Clinical Characteristics Associated With NPY Levels

The distribution of NPY levels in the cohort is shown in eFigure 1 in the Supplement (mean [SD], 85.1 [31] pg/mL; eResults in the Supplement). We examined whether relevant clinical characteristics were associated with NPY levels. As shown in Table 2, NPY levels are significantly greater in women, patients with diabetes (especially insulin controlled), and patients with hypertension (especially those taking hydralazine). Renal function, cardiac structural abnormalities, and 6-minute hall walk distance also were significantly associated with CS NPY levels (eFigure 2 in the Supplement). There was no association between NPY levels and NYHA functional class, ischemic cardiomyopathy, prior coronary artery bypass grafting surgery, or prior myocardial infarction (MI) (Table 2).

**Table 2.** Categorical Factors Associated With NPY Levels

Variable	Mean (SD)		*P* Value
	Yes	No	
Male	81.6 (30.1)	97.5 (31.8)	.03^a^
ICM	86.4 (35.5)	83.8 (25.6)	.99
Prior MI	83.3 (30.7)	86.6 (31.5)	.56
Prior CABG	90.0 (40)	82.6 (24.5)	.67
Atrial fibrillation	86.8 (38.8)	84.0 (25.1)	.84
Hyperlipidemia	86.5 (33.6)	81.7 (24)	.52
Hypertension	89.4 (33.8)	73.2 (16.9)	.01^a^
Prior tobacco use	86.1 (30.5)	83.9 (31.9)	.34
β-Blocker use	85.4 (31.1)	82.1 (31.1)	.67
Antiarrhythmic drug use	86.0 (42)	84.9 (28.3)	.54
Prior valve surgery	88.8 (42.4)	84.8 (30)	.95
Type 2 diabetes	96.5 (37.7)	78.9 (24.9)	.009^a^
Hydralazine use	129.2 (51.1)	82.9 (28.3)	.008^a^
Statin use	87.7 (32.8)	78.7 (25.6)	.08
Insulin use	104.8 (26)	81.8 (30.7)	.001^a^
Diuretic use	87.2 (33.7)	78.4 (19.5)	.40
NYHA functional class			
II	71.3 (11.4)	NA	.33
III	81.2 (29.1)	NA	
IV	63.3 (13.6)	NA	

Abbreviations: CABG, coronary artery bypass grafting; ICM, ischemic cardiomyopathy; MI, myocardial infarction; NA, not applicable; NPY, neuropeptide Y; NYHA, New York Heart Association.

^a^ Indicated statistical significance at *P* <.05.

### Coronary Sinus NPY Level and Clinical Outcomes

**Figure 1.**
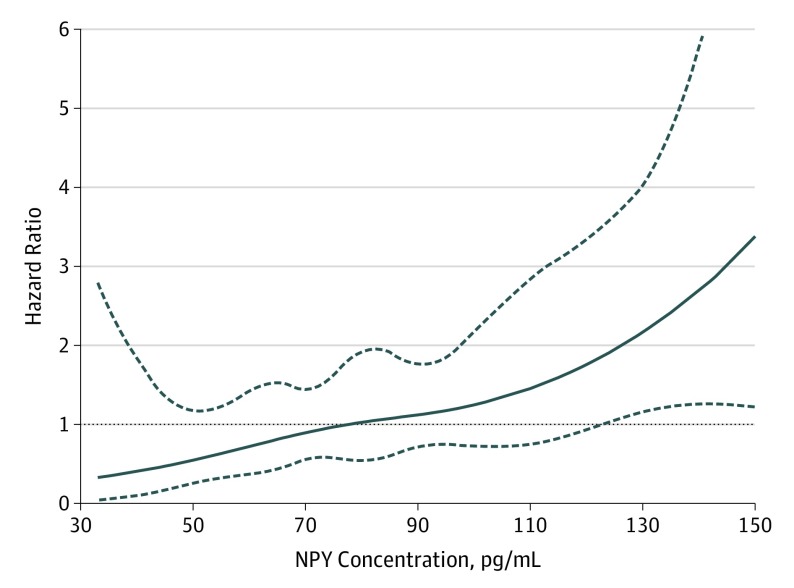
Association Between Coronary Sinus Neuropeptide Y (NPY) Level and Outcome Hazard Ratio (HR) in the Cohort The hazard ratio (solid line) and the upper and lower 95% confidence limits (dashed lines) for major adverse cardiac events (MACE), outcome of death, ventricular assist device placement, and heart transplant and heart failure hospitalization are shown for patients in the cohort (n = 105) after adjusting for age, renal function (glomerular filtration rate), and ejection fraction (LVEF).

**Figure 2.**
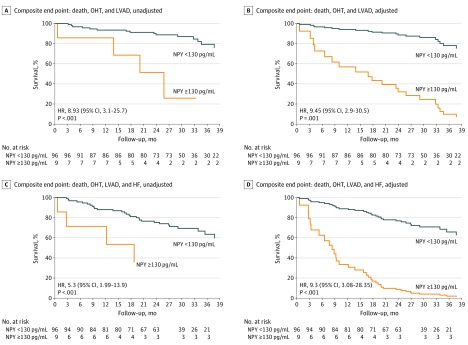
Coronary Sinus Neuropeptide Y (NPY) Levels and Major Adverse Cardiac Events (MACE) A and B, Kaplan-Meier survival analysis for MACE (death, heart transplantation [OHT], or ventricular assist device [VAD] placement) as a function of NPY before (A) and after (B) adjusting for age (hazard ratio [HR], 0.998; 95% CI, 0.95-1.05; *P* = .93), estimated glomerular filtration rate (eGFR) greater than 45 mL/min compared with less than 45 mL/min (HR, 0.325; 95% CI, 0.11-0.96; *P* = .04), and left ventricular ejection fraction (LVEF) per 1% increase (HR, 0.93; 95% CI, 0.86-1.01; *P* = .07), with subanalysis into groups with NPY levels 130 pg/mL or less and NPY levels greater than 130 pg/mL. C and D, Kaplan-Meier survival analysis for MACE (death, OHT, VAD placement, or heart failure hospitalization) as a function of NPY before (C) and after (D) adjusting for age (HR,  0.943; 95% CI, 0.91-0.98; *P* = .001), eGFR greater than 45 mL/min vs less than 45 mL/min (HR, 0.099; 95% CI, 0.038-0.258; *P* < .001), and LVEF per 1% increase (HR, 0.92; 95% CI, 0.88-0.97; *P* = .002), with subanalysis into groups, with NPY level of 130 pg/mL or less and NPY level greater than 130 pg/mL. HF indicates heart failure.

During a median follow-up of 28.8 months, the composite end point of death, OHT, and VAD placement occurred in 20 of 105 patients (19%). A threshold level of greater than 130 pg/mL of CS NPY concentration identified an inflection point at which HR for the composite outcome increased significantly (Figure 1). Patients with CS NPY levels greater than 130 pg/mL had worse outcomes compared with those with lower CS NPY levels (HR, 8.9; 95% CI, 3.1-25.7; *P* < .001). These results were similar after adjusting for age, eGFR, and LVEF (HR, 9.5; 95% CI, 2.92-30.5; *P* < .001) as shown in Figure 2A and B. This was driven predominantly by death (18 events), more than heart transplantation (1 event), or LVAD placement (1 event). The C statistic for MACE was 0.748 (0.04).

The risk of an adverse event remained high when HF hospitalization was added to the composite end point (unadjusted HR, 5.3; 95% CI, 1.99-13.9; *P* < .001). After adjusting for covariates including age, eGFR, and LVEF, the results were similar (HR, 9.34; 95% CI, 3.08-28.35; *P* < .001) as shown in Figure 2C and D. This outcome was driven predominantly by HF hospitalization (28 events) and deaths (8 events). The C statistic for MACE and HF hospitalization was 0.771 (0.044). Of 98 patients who successfully underwent CRT device implantation and had complete follow-up data, 59 were classified as CRT responders based on clinical and echocardiographic changes at 6 months of follow-up. Baseline CS NPY levels did not significantly differ between responders and nonresponders (81.5 [26.3] pg/mL vs 83.7 [27.8] pg/mL; *P* = .76) as shown in the eTable 1 in the Supplement.

### NPY Content in Cardiac Sympathetic Neurons

**Figure 3.**
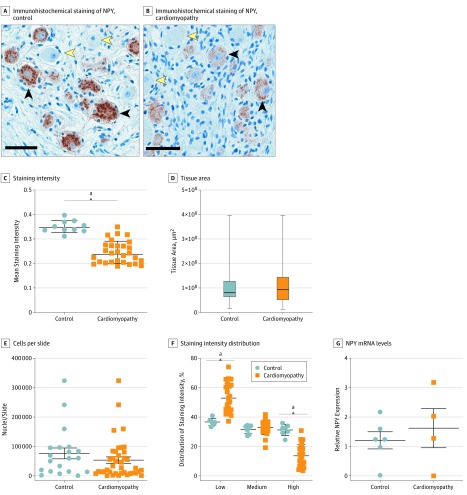
Neuropeptide Y (NPY) Content in Human Stellate Ganglia Immunohistochemical staining of NPY in stellate ganglia from control patients (organ donor) and patients with cardiomyopathy shows reduced NPY immunoreactivity (A) and an overall decrease in staining intensity, measured as optical density (B). This difference was not associated with tissue area compared (C) or number of cells (neurons and glia) per slide (D). Mean staining intensity was decreased in patients with cardiomyopathy (E). Quantitative polymerase chain reaction for NPY mRNA levels (normalized to glyceraldehyde 3-phosphate dehydrogenase) showed no change in NPY messenger RNA (mRNA) in patients with cardiomyopathy compared with controls (F). ^a^*P* < .001. Scale bar: 50 μm.

Postganglionic efferent sympathetic neurons innervating the heart have their soma in the stellate and middle cervical ganglion. To examine whether NPY levels are associated with neuronal NPY content, we compared stellate ganglia from patients with CHF undergoing cardiac sympathetic denervation to those organ donors with structurally normal hearts. Baseline characteristics for these patients are shown in eTable 2 in the Supplement. Neuropeptide Y is stored in dense core vesicles, which are readily appreciated in control patients (Figure 3A and B). Neuropeptide Y immunoreactivity was significantly decreased in patients with CHF compared with control patients, despite similar tissue area examined and cell count per slide (Figure 3C and D). Further classification of the distribution of NPY staining intensity (Figure 3E) revealed that the intensity of staining was evenly distributed across control ganglia, while patients with HF exhibited a shift in staining, where a greater percentage of neurons had low staining intensity, indicating that ganglia from patients with CHF contain less NPY. To examine whether the lower NPY content in CHF ganglia was associated with decreased NPY production, we examined relative NPY mRNA levels. As illustrated in Figure 3F, relative neuronal NPY/glyceraldehyde 3-phosphate dehydrogenase mRNA was similar in patients with CHF and control patients, suggesting no difference in NPY expression.

## Discussion

The main findings of this study are:

1. Coronary sinus NPY levels are associated with specific clinical characteristics including renal function, LV dimensions, and 6-minute walk test distance.
2. Coronary sinus NPY levels are associated with the risk of death, OHT, VAD placement, and HF hospitalization, (albeit in a nonlinear fashion), even when adjusting for age, renal function, and LVEF.
3. Postganglionic neurons in the stellate ganglia have lower NPY content despite equivalent mRNA expression in patients with CHF vs control patients.

To our knowledge, this represents the first description of these findings in humans.

In animal models and in humans, circulating NPY levels are elevated during acute coronary syndromes,^12^ LV dysfunction,^13,14^ and in CHF.^15,16^ Early studies, before the advent of modern medical and interventional treatment, associated peripheral venous NPY levels with 1 year mortality in patients with acute MI or HF admitted to a coronary care unit.^14^ These studies only measured NPY-like activity, whereas our assay has a very low limit of detection (approximately 3 pg/mL) and high specificity, with 0% cross-reactivity with structurally similar peptides. Moreover, peripheral venous NPY levels are not cardiac specific and predominantly reflect hepatomesenteric release because as NPY has been implicated in stimulating food intake.^17^

While CS NPY levels are associated with catecholamine levels in CHF patients,^15^ its prognostic value is poorly understood. In this prospective observational cohort, mean (SD) CS NPY levels were 85.1 (31) pg/mL (range, 33-213 pg/mL), substantially higher than mean levels observed in a cohort of patients with normal coronary arteries and normal LVEF using the same assay (4.5  [2.5] pg/mL).^18^ Although transcardiac NPY levels were not assessed, NPY spillover in the cardiac vascular bed is increased in heart failure, as demonstrated by Morris et al.^16^ Specifically, CS levels NPY concentration was higher than that in arterial blood in patients with HF at rest, suggesting that cardiac release is a significant source of NPY in HF. Therefore, in this study, we have measured CS NPY, which is more reflective of cardiac NPY release than peripheral levels, using an assay that is specific for NPY and not related peptides.

### Coronary Sinus NPY Levels and Clinical Indices

Coronary sinus NPY concentration was associated with several clinical factors associated with HF symptoms or with prognostic implications in patients with HF. While the mechanism of elimination is not well understood,^16^ plasma NPY levels are elevated in patients with renal dysfunction.^19,20^ In accordance, CS NPY levels were associated with eGFR, serum blood urea nitrogen, and creatinine levels. Additionally, LV and left atrial dimensions also inversely associated with CS NPY concentration, indicating that CS NPY levels are reduced as cardiac dilatation worsens. There was no association between LVEF and CS NPY level, suggesting that the association with cardiac dilatation is not necessarily related to LV function. This finding may indicate a reduction in cardiac NPY release in severely dilated hearts and/or an overall reduction in innervation, supported by the risk imparted by a reduced heart to mediastinal ratio and iobenguane washout in the ADMIRE-HF study.^21^ Coronary sinus NPY levels were also associated with the 6-minute walk test, a prognostic factor in patients with HF, and with N-terminal–pro hormone brain natriuretic peptide levels, a marker of HF symptoms^22^ and risk of HF hospitalization.^23^

### Clinical Implications

Severely elevated CS levels of NPY (>130 pg/mL) at CRT implantation were associated with MACE (death, heart transplant, LV assist device placement, and HF hospitalization). This suggests a threshold association between CS NPY levels and MACE, and severely elevated CS NPY levels are prognostic. Importantly, CRT response was not associated with baseline CS NPY levels. Because NPY release is associated with adrenergic tone, levels greater than 130 pg/mL likely severe adrenergic excess and neurohormonal activation, which in turn have been associated with worse clinical outcomes,^1^ including pump failure.^24^ This is supported by Cohn et al,^6^ who demonstrated higher norepinephrine levels in patients who died of pump failure.

To explore the mechanisms for elevated CS NPY levels, we performed immunohistochemistry on stellate ganglia neurons because it provides the bulk of the postganglionic sympathetic innervation to the heart and is an important source of NPY. For example, following MI in the pig, NPY immunoreactivity in the stellate ganglia increases.^25^ The reduction in immunoreactivity seen in neurons from patients with CHF in our study was not associated with decreased production of NPY as suggested by quantitative polymerase chain reaction. We infer from these findings that transport to distal axonal endings and increased release in patients with cardiomyopathy contributes to the higher CS NPY levels seen in these patients compared with control patients (eFigure 3 in the Supplement). Hence, a component of adrenergic remodeling that occurs in chronic HF is increased axonal transport and release into cardiac tissue beds, accounting for elevated levels observed in this study.

Coronary sinus NPY levels may identify patients in whom close clinical monitoring and more aggressive interventions are needed to prevent adverse events. It may also identify those in whom CRT is likely to be ineffective, and such patients may be considered sooner for OHT or VAD. More importantly, elevated circulating NPY in patients with HF may contribute to the complex pathophysiology of chronic HF and promote LV dysfunction. Our findings warrant further mechanistic studies in animal models and in humans (eg, using mendelian randomization approaches) to establish a causal effect for NPY in HF progression. Antagonism of NPY signaling (given its potentiating actions on adrenergic signaling) may mitigate progressive HF beyond current guideline-directed pharmacotherapy.

### Limitations

Transcardiac release or spillover or peripheral venous levels were not assessed in this study; hence, cardiac or systemic NPY release could not be directly quantified and distinguished. Prior studies of NPY release across multiple vascular beds demonstrate that CHF increases cardiac NPY spill over significantly.^16^ Further, hepatomesenteric release provides a major contribution to circulating NPY levels, making CS sampling a more accurate reflection of cardiac NPY release in CHF. All patients in this study underwent CRT implantation. Although CS NPY levels were not associated with CRT response, the presence of CRT devices likely affected the study’s findings and limits its applicability to the CHF population undergoing CRT. In this study, we did not measure indices of adrenergic function and are unable to associate NPY level with cardiac adrenergic tone. Given the limited number of patients with CS NPY levels greater than 130 pg/mL, the hazard ratios may overestimate the risk associated with elevated NPY levels. Last, the sample size of 105, while robust in terms of CS blood sampling, did not allow for formal statistical validation of these findings, including the NPY thresholds. Validation should be carried out in future studies.

## Conclusions

We demonstrate for the first time, to our knowledge, in this prospective observational study that CS NPY levels are elevated, associated with adverse outcomes, and are significantly associated with clinical and laboratory characteristics in patients stable CHF. Increased stellate ganglia neuronal release is likely responsible for the elevated levels. These data suggest that CS NPY levels may provide prognostic information in patients with CHF. Larger studies are warranted to confirm these findings.

## Notes

Supplement. eMethods. **eResults. ** **eFigure 1.** Histogram of Coronary Sinus NPY values in patients undergoing CRT device implantation **eFigure 2.** Coronary sinus NPY levels correlate with severity of renal dysfunction and cardiac remodeling **eFigure 3.** Summary figure of Neuropeptide-Y in chronic heart failure **eTable 1.** Relationship between NPY Concentration and Response to Cardiac Resynchronization Therapy (CRT) **eTable 2.** Baseline characteristics of stellate ganglia donors
