# Supplementary material for: Coronary Sinus Neuropeptide Y Levels and Adverse Outcomes in Patients With Stable Chronic Heart Failure
Source: JAMA Cardiol. 2019 Dec 26;5(3):318–25. doi: 10.1001/jamacardio.2019.4717 (PMC6990798; doi:10.1001/jamacardio.2019.4717)
Supplement: Supplement. — eMethods. eResults. eFigure 1. Histogram of Coronary Sinus NPY values in patients undergoing CRT device implantation eFigure 2. Coronary sinus NPY levels correlate with severity of renal dysfunction and cardiac remodeling eFigure 3. Summary figure of Neuropeptide-Y in chronic heart failure eTable 1. Relationship between NPY Concentration and Response to Cardiac Resynchronization Therapy (CRT) eTable 2. Baseline characteristics of stellate ganglia donors [file jamacardiol-5-318-s001.pdf]

Supplementary Online Content

Ajijola OA, Chatterjee NA, Gonzales MJ, et al. Coronary sinus neuropeptide Y levels and adverse outcomes in patients with stable chronic heart failure. Published online December 26, 2019. *JAMA Cardiol*. doi:10.1001/jamacardio.2019.4717

eMethods.
eResults.
eFigure 1. Histogram of Coronary Sinus NPY values in patients undergoing CRT device implantation
eFigure 2. Coronary sinus NPY levels correlate with severity of renal dysfunction and cardiac remodeling
eFigure 3. Summary figure of Neuropeptide-Y in chronic heart failure
eTable 1. Relationship between NPY Concentration and Response to Cardiac Resynchronization Therapy (CRT)
eTable 2. Baseline characteristics of stellate ganglia donors

This supplementary material has been provided by the authors to give readers additional information about their work.

© 2019 Ajijola OA et al. *JAMA Cardiology*.

Supplemental Methods

Study population and device implantation

Study approval was obtained from the Massachusetts General Hospital institutional review board. Data were obtained from patients enrolled in the prospective, single center, observational, Biomarkers to Predict CRT Response in Patients with CHF (BIOCRT; NCT01949246) study. Inclusion criteria included patients meeting guideline indications for CRT implantation (New York Heart Association, NYHA, class II-IV heart failure refractory to optimal drug therapy, left ventricular ejection fraction, LVEF < 35%, and QRS width > 120ms); concurrent use of optimal drug therapy, and with decompensated CHF in the prior 12 months. The study excluded patients with NYHA class I functional score, severe aortic stenosis, cardiac surgery with prior 90 days, severe obstructive pulmonary disease requiring oxygen or with recent decompensation (<30 days), current pregnancy, primary pulmonary hypertension, continuous intravenous drug infusion for heart failure, and life expectancy under 6 months.

Pre- procedure demographics, biometrics, and clinical evaluation including detailed medical history, NYHA functional class, six-minute walk test, Minnesota living with heart failure questionnaire score, twelve-lead electrocardiography, and two-dimensional transthoracic echocardiography (TTE) were performed. Serum creatinine, blood urea nitrogen, and N-terminal pro-brain natriuretic peptide (NT-ProBNP) levels were measured. Following device implantation, routine clinic visits at 1, 3, and 6 months were performed, and data were collected for a period of two years. The Social Security Death Index was examined to determine the date

© 2019 Ajijola OA et al. *JAMA Cardiology.*

of death.

Stellate ganglia immunohistochemistry

The study was approved by the UCLA institutional review board and written informed consent by the patient or appropriate designee obtained. Stellate ganglia from controls (organ donors at the time of heart/lung organ procurement, n=4) with structurally normal hearts, and CHF patients undergoing cardiac sympathetic denervation (the resection of the lower half of both SG, and second through fourth paravertebral ganglia, n=13) were collected, fixed in formalin, and paraffin embedded. Sections 5µm thick were taken for immunostaining with antiserum to NPY (ab112473; 1:2000 dilution). Diaminobenzidine reaction (Life Technologies,) was used for detection. Slides were scanned (Scanscope, Aperios Systems) for digital analysis (Tissue Studio, Definiens Inc.).

Stellate ganglia quantitative polymerase chain reaction

Stellate ganglia samples were collected from patients with CHF undergoing cardiac sympathetic denervation (n=4 from 2 patients), or from organ donors (n=6 from 3 patients) with structurally normal hearts at the time of organ procurement. Two technical repeats were performed per patient (one form each stellate ganglia). Total RNA from human tissue was extracted using an RNeasy Protect mini kit (Qiagen). For reverse transcription, first-strand cDNA was synthesized from 1 µg of total RNA with the iScript™ cDNA Synthesis kit (Biorad). Quantitative polymerase chain reaction was conducted in a total of 20 µl containing 10 µl of Taqman Universal PCR Master mix (Applied Biosystems), 4 µl of cDNA (10ng/µl), 1 µl of 20X specific primers for Taqman Gene Expression Assays (Hs00173470_m1 for human NPY,

© 2019 Ajijola OA et al. *JAMA Cardiology.*

Hs02786624_g1 for human GAPDH, Thermo Fisher Scientific) and 5 µl of DNase-free water. Quantitative real-time RT-PCR was performed in a 96-well clear optical reaction plate (Applied Biosystems), and thermal cycling conditions were: 2 min at 50°C, 10 min at 95°C, followed by 40 cycles of 15 s at 95°C and 1 min at 60°C. Results were analyzed with the ABI Prism 7000 Sequence Detection System software (Applied Biosystems). Gene expression was normalized to GAPDH.

Statistical analysis

Bivariate relations of NPY with clinical variables

Continuous variables are reported as mean ± standard deviation (SD) or median with interquartile range if not normally distributed, and nominal variables as frequency or percentages. The p values for mean NPY comparisons across categorical or two group (binary) predictors were computed using the t-test since NPY was normally distributed. NPY immunoreactivity in control and cardiomyopathy groups was compared using the Mann-Whitney test for non-normally distributed data. The association between continuous predictors such as age and NPY was assessed using the Spearman correlation (r_s), and by simple linear regression. Linearity of relationship between a continuous potential predictor versus NPY was assessed by comparing the linear model to a restricted cubic spline fit for the same predictor via a likelihood ratio test.

NPY versus MACE

© 2019 Ajijola OA et al. JAMA Cardiology.

The relationship between CS NPY and time to MACE was assessed by considering NPY as a continuous predictor using restricted cubic splines (RCS) in a Cox proportional hazard regression and by searching for a critical NPY threshold that best separated low from high MACE hazard. This NPY threshold was estimated using a tree structured survival analysis using MACE hazard as the outcome and carrying out binary recursive partitioning. The hazard ratio (HR) and its 95% confidence interval limits (CI) and corresponding p value are reported. Once the critical NPY threshold was determined, the effect of NPY was assessed both ignoring and controlling for the critical covariates of age, reduced glomerular filtration rate and LVEF using a Cox proportional hazard model.

Supplemental Results

Clinical characteristics predictive of NPY levels

The distribution of NPY levels in the cohort is shown in efigure 1 (mean 85.1±31pg/ml). We examined whether relevant clinical characteristics were related to NPY levels. As illustrated in efigure 2, higher CS NPY levels were seen as estimate glomerular filtration rate (eGFR) decreases (r_s= -0.36, p=0.0002), and blood urea nitrogen (r_s=0.30, p=0.0018) and serum creatinine levels increase (r_s=0.22, p=0.023). Interestingly, CS NPY was negatively correlated with LV internal dimensions in diastole (r_s= -0.35, p=0.0004) and systole (r_s= -0.30, p=0.0033), and with left atrial diameter (r_s = -0.23, p=0.022). There was no association between LVEF and NPY levels (r_s = 0.06, p=0.54). Baseline six-minute walk test (6MWT) distance, an objective measure of functional status of patients, and NT-ProBNP levels, a biomarker for HF symptoms and prognosis correlated with NPY levels (r_s= -0.32, p=0.012, and r_s = 0.33, p=0.008, respectively).

© 2019 Ajijola OA et al. JAMA Cardiology.

Supplemental Figure Legends

eFigure 1. Histogram of Coronary Sinus NPY values in patients undergoing CRT device implantation.

eFigure 2. Coronary sinus NPY levels correlate with severity of renal dysfunction and cardiac remodeling.

Correlation between CS NPY levels and several clinical, laboratory, and echocardiographic characteristics are shown with 95% CI. NPY: Neuropeptide Y; BUN: blood urea nitrogen; Cr: creatinine; eGFR: estimated glomerular filtration rate; LVIDd: left ventricular internal diameter in diastole; LVIDs: left ventricular internal diameter in systole; 6MWT: six-minute walk test.

eFigure 3. Summary figure of Neuropeptide-Y in chronic heart failure

One-sentence Summary: The adrenergic co-transmitter neuropeptide Y is associated with adverse outcomes in stable heart failure patients.

© 2019 Ajijola OA et al. *JAMA Cardiology.*

Supplemental Table

eTable 1. Relationship between NPY Concentration and Response to Cardiac Resynchronization Therapy (CRT).

	CRT-responder				
NPY (pg/ml)	No	Yes		CRT Responder (%)	*p* value
< 130	56	36		39.1%	0.9230
>= 130	3	3		50.0%	

eTable 2. Baseline characteristics of stellate ganglia donors

Group	Age	Gender	BMI	Etiology	LV EF	NYHA	CAD	Arrhythmia	HTN	Diabetes	eGFR
**Cardiomyopathy**	49	M	25.7	NICM	30%	II	No	VT	No	No	95
**Cardiomyopathy**	66	M	30.2	NICM	50%	II	No	VT/VF	No	Yes	66
**Cardiomyopathy**	74	M	25.1	NICM	15%	II	No	VT	Yes	No	64
**Cardiomyopathy**	55	F	20.3	ICM	40%	II-III	Yes	VT	Yes	Yes	HD
**Cardiomyopathy**	50	F	21.8	NICM	45%	II	No	VT	Yes	No	94
**Cardiomyopathy**	54	M	24.4	NICM	22%	II	No	VT	Yes	No	94
**Cardiomyopathy**	31	M	18.7	NICM	10%	III-IV	No	VT	No	No	50
**Cardiomyopathy**	64	F	34.1	ICM	20%	III	Yes	VT	Yes	Yes	40
**Cardiomyopathy**	43	M	37.6	NICM	55%	II	No	VT/VF	No	No	107
**Cardiomyopathy**	68	M	29.3	NICM	56%	II-III	No	VT	No	No	93
**Cardiomyopathy**	46	F	26.6	n/a	50%	I	No	VT/VF	No	No	96
**Cardiomyopathy**	69	M	29.6	NICM	30%	II	Yes	VT	Yes	No	89
**Cardiomyopathy**	18	F	17.5	NICM	50%	I	No	VT/VF	No	No	116
**Cardiomyopathy**	76	M	28.6	NICM	30%	II	No	VT/VF	Yes	No	62
**Cardiomyopathy**	62	M	36.6	NICM	47%	II	Yes	PMVT/VF	Yes	No	78
**Control**	48	F	35.4	n/a	55%	I	No	n/a	No	No	31
**Control**	30	M	23.9	n/a	70%	I	No	n/a	No	No	119
**Control**	48	M	30	n/a	65%	I	No	n/a	Yes	No	41
**Control**	63	F	22.5	n/a	65%	I	No	n/a	Yes	Yes	44
**Control**	61	F	27.1	n/a	65%	I	No	n/a	Yes	No	20
**Control**	19	M	26.2	n/a	60%	I	No	n/a	No	No	72

BMI: body mass index; CAD: coronary artery disease history; eGFR: estimated glomerular filtration by Modification of Diet in Renal Disease (MDRD) Study; HTN: hypertension; ICM: ischemic cardiomyopathy; LV EF: left ventricular ejection fraction; NICM: nonischemic cardiomyopathy; NYHA: New York Heart Association functional class; PMVT: polymorphic ventricular tachycardia; VF: ventricular fibrillation; VT: ventricular tachycardia.

© 2019 Ajijola OA et al. *JAMA Cardiology.*

Supplemental Figures

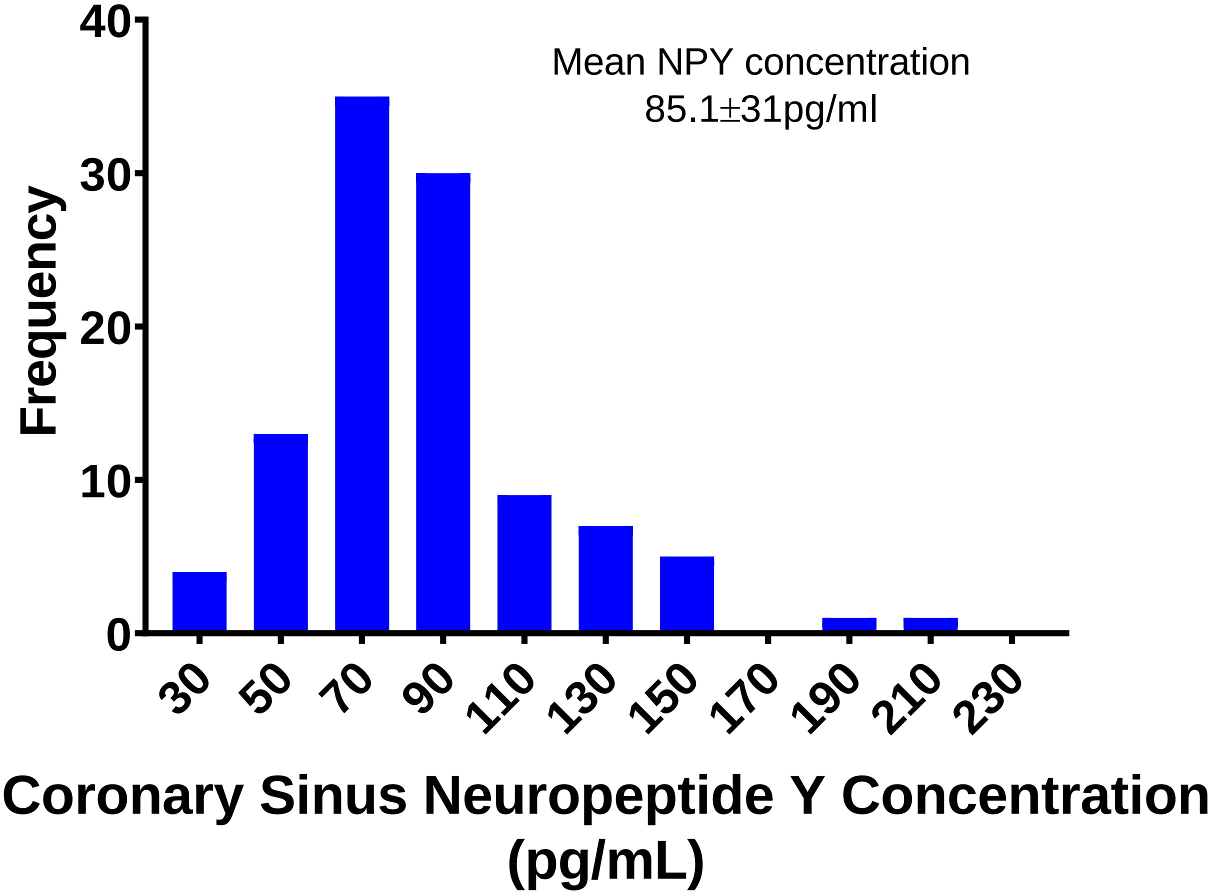

eFigure 1. Histogram of coronary sinus NPY values in study patients.

© 2019 Ajijola OA et al. *JAMA Cardiology.*

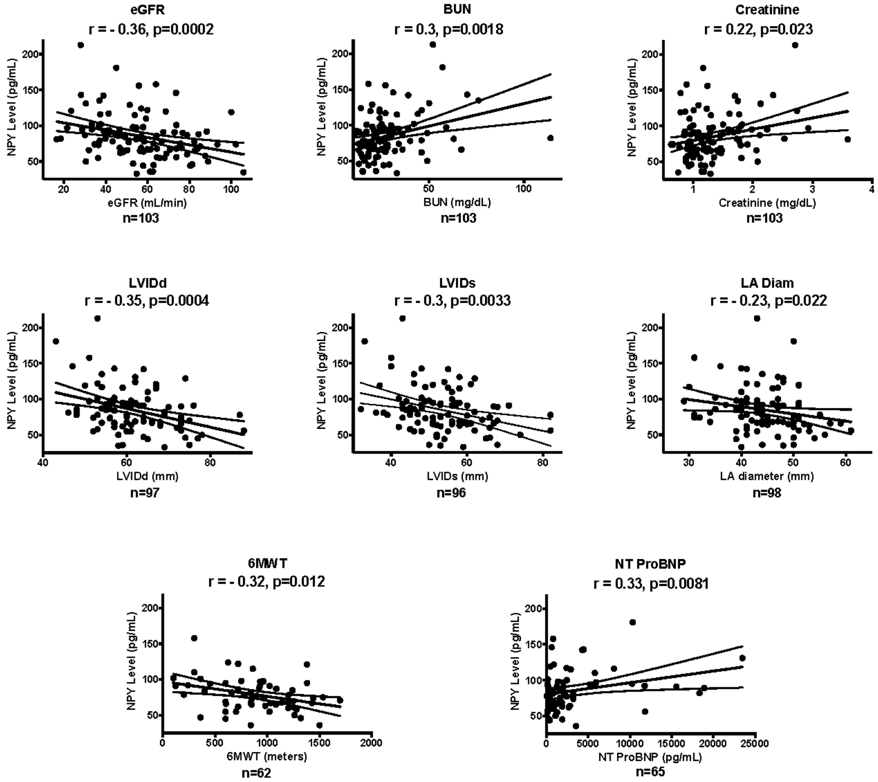

eFigure 2. Coronary sinus NPY levels correlate with severity of renal dysfunction and adverse cardiac remodeling

© 2019 Ajijola OA et al. *JAMA Cardiology.*

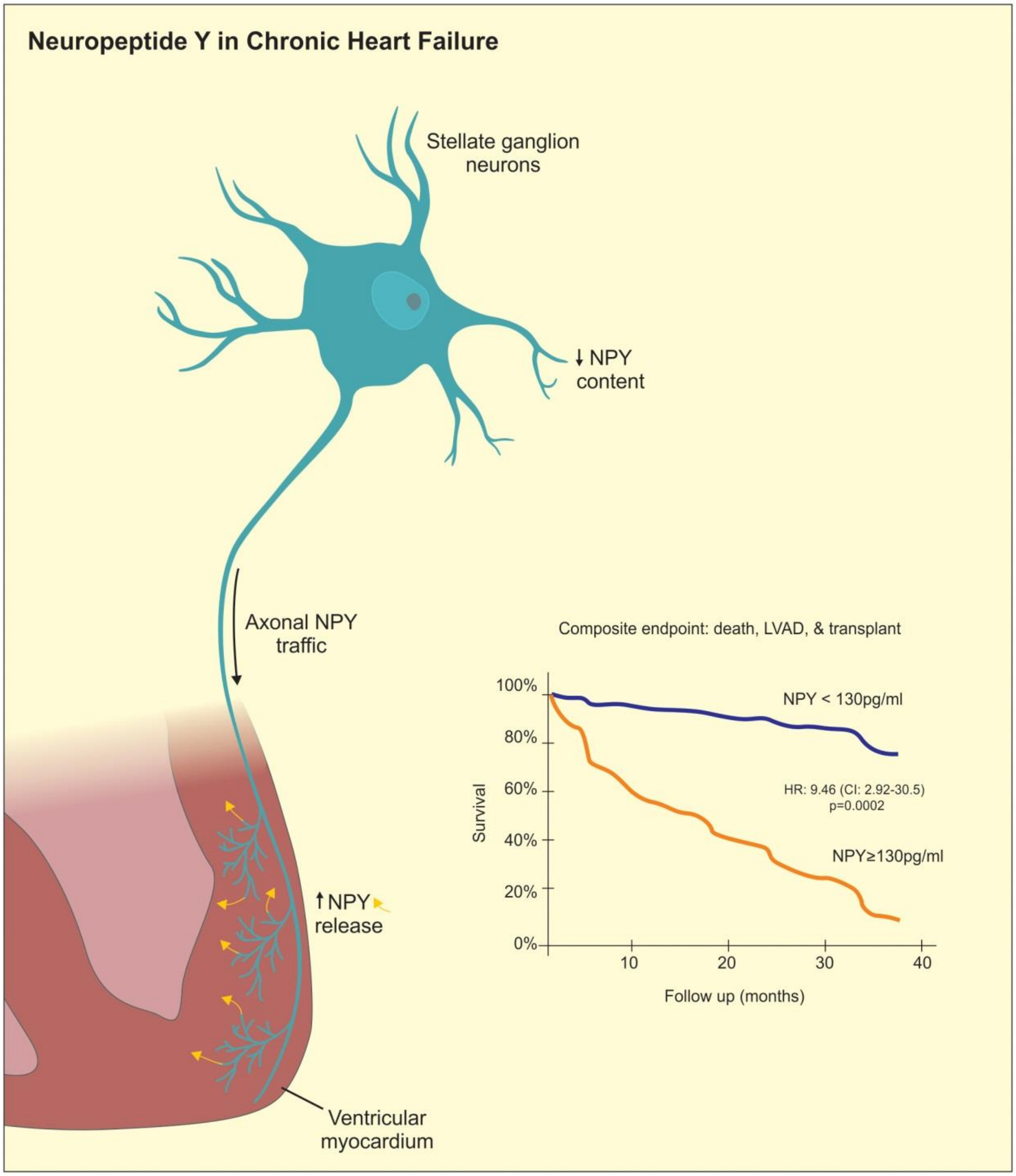

eFigure 3. Summary Figure of study findings.

© 2019 Ajijola OA et al. *JAMA Cardiology*.
